# The impact of testing-parameter variability on force production in the isometric single-leg long-lever bridge: implications for training and testing rigor in sporting environments

**DOI:** 10.1186/s13102-025-01407-9

**Published:** 2025-11-25

**Authors:** Adam E. Sundh, Nicholas J. Ripley, AJ Lamb, Conor J. Cantwell, Paul Comfort

**Affiliations:** 1 School of Health and Society, University of Salford, Salford, M5 WT UK; 2 Chicago Bears Football Club, 1920 Football Drive, Lake Forest, IL 60045 USA; 3 Department of Athletics, William & Mary, Williamsburg, VA 23187 USA; 4 School of Medical and Health Sciences, Edith Cowan University, Joondalup, 6027 Australia

**Keywords:** Isometric strength, Performance monitoring, Hamstring assessment, Long-lever bridge, Force plates

## Abstract

**Background:**

The aim of this study was to determine the impact of knee angle variability on force production outcomes during the single-leg isometric long-lever bridge, thus providing monitoring guidelines for testing rigor with direct implications for feasibility across a variety of high-performance sporting environments.

**Methods:**

Thirty men (age: 19.4 ± 1.3 years; height: 179.8 ± 6.3 cm; body mass: 80.4 ± 10.3 kg) and 14 women (age: 20.0 ± 1.3 years; height: 166.9 ± 7.2 cm; body mass: 64.4 ± 7.4 kg) all of whom were Division 3 athletes with no recent injury history volunteered to participate in the study. All participants completed three testing sessions over five days in randomized order, with knee flexion angles of 15°, 30°, or 45° degrees. Each session consisted of three unilateral maximal voluntary isometric contractions (MVIC), with the athlete’s heels positioned on force plates, shoulders elevated on a 15.24 cm box, and hips secured using a rigid barbell. All data was analyzed to assess net force production comparisons between knee angles at 50, 100, 150, 200, 250 ms and peak force (N).

**Results:**

Large variance in force outputs were observed ranging between 0.30 and 0.82 (male *η*_*p*_*²*), and 0.52–0.76 (female *η*_*p*_*²*), and significant differences observed between all knee angles (*p* < 0.05). Pairwise comparisons revealed effect sizes ranging between small-large for males (Hedge’s *g* = 0.27–1.84) and moderate to very large for females (*g* = 0.51–2.18) across individual force-time points.

**Conclusions:**

These findings indicate that the knee angle must be rigorously controlled when performing the single-leg isometric long-lever bridge to mitigate unwanted variability and to accurately assess the intended musculature, especially for longitudinal monitoring.

## Introduction

Emerging research findings highlight the increased popularity in monitoring isometric hamstring strength [1–5], likely due to the large incidence of hamstring strain injuries (HSI) in field-based sports [6]. HSI’s along with several other non-contact injuries, have multiple non-modifiable risk factors such as age and previous HSI [7]. However, the two main modifiable risk factors appear to be fascicle length of the biceps femoris long head (BF_LH_) [8] as well as hamstring strength [7]. More specifically, eccentric hamstring strength is perhaps the most common hamstring strength assessment strategy leveraged in sport [8–11]. Nonetheless, hamstring strength assessments in general may be considered more feasible in practice when compared to measuring fascicle lengths, as accurately measuring the fascicle length of the BF_LH_ requires specialized expertise in ultrasonography. However, although such assessments provide a practical means of assessing overall hamstring function, they do not allow for verification of the exact force produced by individual hamstring musculature, as fascicle length measurements do for each muscle. Considering various factors like strength and fatigue are variable [7], it may not be appropriate to assess these qualities on a single occasion. Instead, it might be better to continually monitor the changes in force production characteristics that the individuals demonstrate, as recommended by Opar et al. [9], to ensure a comprehensive assessment and allowing for the evaluation of any changes in risk factors over time. This ongoing monitoring allows the identification of potential issues such as increased fatigue or reduced strength due to detraining and therefore provides the opportunity to make timely adjustments in programming and load monitoring strategies. Thus, by assessing these risk factors longitudinally, a clearer picture of individual progress or potential risk dynamics can be achieved considering the risk factors may change over the course of a competitive season [12].

To appropriately monitor an athletes’ hamstring strength longitudinally, the assessment must be both valid and reliable within the specific cohort in which the assessment is being performed [13]. Recent advancements in field-based assessment tools such as force plates, hand-held dynamometers, strain gauges and the Nordbord amongst many others, have minimized many of the typical limitations of laboratory-based equipment like isokinetic dynamometry, namely high costs, lack of portability, limited accessibility, and time-consuming protocols. As a result, with both more portable and affordable solutions, the ability to assess hamstring strength may now be much simpler and time-effective in practice. As highlighted by Guthrie et al., [14], tissue-specific assessments, such as those examining individual muscles’ mechanical properties, offer valuable insights into performance and injury risk, further emphasizing the potential of leveraging these technologies in high-performance environments. This is made evident with recent literature assessing both eccentric and isometric hamstring strength specifically with these technologies in field-based environments [9, 15, 16].

With the increasing prevalence of both eccentric and isometric hamstring assessments, prioritizing isometric hamstring strength may prove more feasible due to its minimal impact on delayed onset muscle soreness (DOMS) [17], thus reducing the barriers to implementation. Moreover, recent evidence indicates that isometric hamstring strength can similarly identify athletes at risk of future HSI [18], supporting its use as a valid alternative to eccentric assessments. Before using any longitudinal assessment however, it is important to assess the reliability to ensure the results report real changes in performance rather than measurement error. An important factor to consider is the sensitivity of the assessment to variations in testing parameters, including joint angles, limb positioning, equipment, footwear, and surface characteristics. The aforementioned factors may influence the outcome of the assessment by altering force application, stability, and introducing unintended variations due to uncontrolled or non-considered elements. These influences may lead to discrepancies in the results thereby compromising the consistency and reproducibility of the assessment due to unfamiliar conditions. In other isometric tests, such as the isometric mid-thigh pull (IMTP), joint angles have been shown to substantially impact force production [19], likely reflecting mechanical factors, such as lever-arm configuration and perhaps changes in the length-tension relationship [20], although the latter is challenging to confirm directly. In the context of longitudinal monitoring, this concern becomes even more critical, as the assessment protocol must remain consistent with the initial evaluation to ensure that any observed change in performance is not attributable to any discrepancy in the testing procedure. Failure to maintain methodological consistency between assessments may lead to erroneous interpretations where perceived changes in performance are a result of procedural inconsistencies rather than measurable change in performance.

Previous researchers investigating isometric hamstring assessments have displayed a change in muscle activation with various knee [21, 22] and hip [23] angles highlighting the importance of ensuring the proper hamstring musculature is evaluated during the assessment. Thus, as knee angle increases, it is reasonable to expect that a change in emphasis may occur away from the BF_LH_ [24], however the semimembranosus may follow a similar pattern [25]. Consequently, force production at specific knee angles may not reliably indicate selective recruitment of a particular hamstring muscle. Nonetheless, it may be suggested that a smaller knee angle during the assessment may be preferrable to reduce the risk of shifting the emphasis to the other musculature. Although this approach does not isolate the BF_LH_, it may provide a practical alternative by assessing force production at joint angles that are more relevant to common injury mechanisms. As such, erring on the side of caution by using a smaller knee angle may be a prudent approach for assessing posterior chain musculature. Therefore, the purpose of this study was to examine the sensitivity to alterations in force production characteristics with a change in knee angle, thus providing monitoring guidelines for testing rigor with direct implications for feasibility across a variety of high-performance sporting environments. By focusing on how force production varies with knee angles, this approach aims to establish a foundation for achieving consistent measurements, which is essential for ensuring reliable assessments in the future. It was hypothesized that the highest amount of force would be produced at a knee angle of 45° degrees of knee flexion and the lowest amount of force experienced at 15° degrees of knee flexion, inferred from previous assessment methods [25]. Additionally, a significant difference in force production was expected across the three knee angle positions (15°, 30°, and 45° of knee flexion).

## Materials and methods

### Participants

A total of 49 participants competing in Division 3 collegiate athletics were initially recruited for the study, including football (American; *n* = 16), men’s soccer (*n* = 14), women’s soccer (*n* = 15) baseball (*n* = 3), and track and field (*n* = 1). However, due to illness (*n* = 1) and scheduling conflicts (*n* = 4), 5 total athletes were unable to complete all sessions. This resulted in a final sample comprising of 30 men (age: 19.4 ± 1.3 years; height: 179.8 ± 6.3 cm; body mass: 80.4 ± 10.3 kg) and 14 women (age: 20.0 ± 1.3 years; height: 166.9 ± 7.2 cm; body mass: 64.4 ± 7.4 kg). To participate in the study, all individuals were required to have had no history of sustained hamstring injuries within the 6 months prior to the study and no catastrophic lower extremity injuries in the 12 months preceding the study. Injuries were defined as any tissue damage that led to missed time from practice or competition, with catastrophic injuries considered as those that resulted in an absence of more than six months. Prior to recruiting participants, organizational consent was obtained from a senior administrator within the athletics department. Participants were provided with detailed information about the study before its commencement and were required to give written informed consent prior to their involvement. Ethical approval for the study was obtained from the University of Salford’s institutional ethics committee (reference no: 0902, approval date: 11 November 2024), in line with the Declaration of Helsinki. An a priori sample size estimation indicated that a minimum of 14 participants were required to achieve a statistical power of 80%, an alpha level of 0.05 and based on detecting, at a minimum, a large difference (*η*_*P²*_ = 0.2), well above the typical threshold for a large effect size (*η*_*P²*_ = 0.14). This estimation is supported by similar findings in knee joint angle interactions, as observed in studies like Hirose et al. [22], where substantially larger effect size variances were noted among the three groups.

### Experimental design

A randomized, within-subject crossover design was used to compare force production characteristics across three joint angles during an isometric posterior chain assessment. Each participant completed all testing conditions, with the order of joint angle exposure randomized to mitigate potential order effects. Participants completed a familiarization session 48 h prior to formal testing to become accustomed to the procedures and minimize potential learning effects. Testing sessions were separated by 48 h to allow for adequate recovery, spanning a total of five days. Data collection occurred during participants’ normal training hours, with each testing session conducted at the same time of day to minimize diurnal variability.

### Isometric single-leg long-lever bridge

**Fig. 1:**
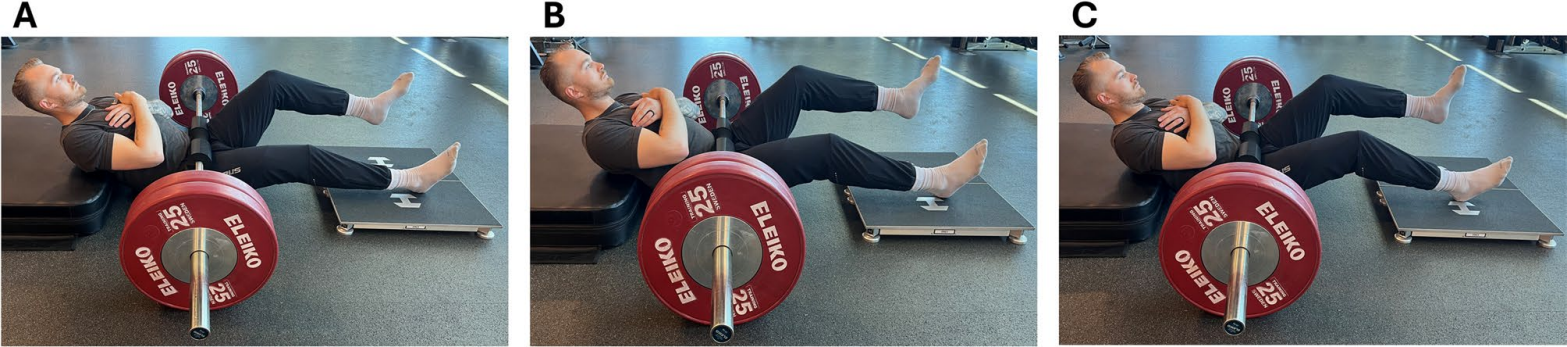
**a–c**. Visual representation of the single-leg long-lever bridge assessment performed at knee angles of (**A**) 15° degrees, (**B)** 30° degrees, and (**C**) 45° degrees

The isometric single-leg long-lever bridge was conducted using Hawkin Dynamics force plates (Hawkin Dynamics Inc., Portland, ME, USA) with a sampling rate of 1000 Hz and data collection via the Hawkin Dynamics mobile software. While participants were positioned supine, the force plates were placed on the ground with direct contact to the heels of the participant and the inferior angle of the scapula placed on top of a 15.24 cm (6 inch) foam platform. This was done to elevate the torso and to facilitate the intent for hip extension during the assessment. A loaded barbell, secured with a rigid foam pad at the inguinal fold was used to anchor the lower extremity and provide a point of resistance for the participants to push against. Each test was then performed unilaterally with the knee set at three different angles of 15°, 30°, and 45° of knee flexion. For the baseline condition, the testing limb was positioned with a knee angle of 30° to emphasize muscle activation of the BF_LH_ [22], as commonly employed in previous research [4, 13, 26], while maintaining a hip angle of 135° ± 5°. The 15° and 45° knee angle conditions were selected to provide equal deviations from the baseline, resulting in approximate hip angles of 145° ± 5° and 125° ± 5°, respectively. This was done to assess the sensitivity of force production to small changes in knee angles, building on Hirose et al. [22], who demonstrated substantial EMG differences with larger 30° knee angle changes. Knee angles were measured using a standard goniometer and were verified before and during each trial to ensure they were maintained throughout the test execution. Finally, the contralateral limb was elevated in the air with the knee bent during each assessment (Fig. 1). During each trial, participants performed the isometric task by driving their heels into the force plates while attempting to extend their hips into the resisted barbell for 3–5 s. Participants were cued to push as hard and fast as possible while driving their heels into the force plates and extending their hips into the loaded barbell. They were further instructed to remain as still as possible for 1 s before each trial commenced, to allow for accurate limb weight and force-time calculation. To ensure accurate depiction of each athletes’ performance, each limb underwent three trials per assessment, with any trial that deviated by ≥ 50 Newtons (N) from the previous one being repeated.

### Data analysis

Force-time data were recorded at a sampling rate of 1000 Hz and processed using Python’s SciPy package. To improve the reliability of the measurements corresponding to rate of force production [27], a low-pass 4th order Butterworth filter with a cutoff frequency of 50 Hz was applied to maximize preservation of the original signal. Onset of movement was classified where force exceeded five standard deviations (SD) above the average force recorded during the one-second weighing phase, consistent with previous methodology [28]. Force values in Newtons (N) were extracted at specific time point, 50 ms, 100 ms, 150 ms, 200 ms, 250 ms, and peak force from the onset of movement until each respective time interval. Net force was determined as the force produced above limb weight (determined from the one-second weighing phase) at the respective time points. Net force was used for analysis to account for variations in system mass and limb weight at different knee angles, as changes in knee angle may generate varying pressure on the force plate, potentially affecting force outputs at early time points. For final analyses, mean force values across the three trials were calculated for both limbs combined.

### Statistical analyses

**Fig. 2:**
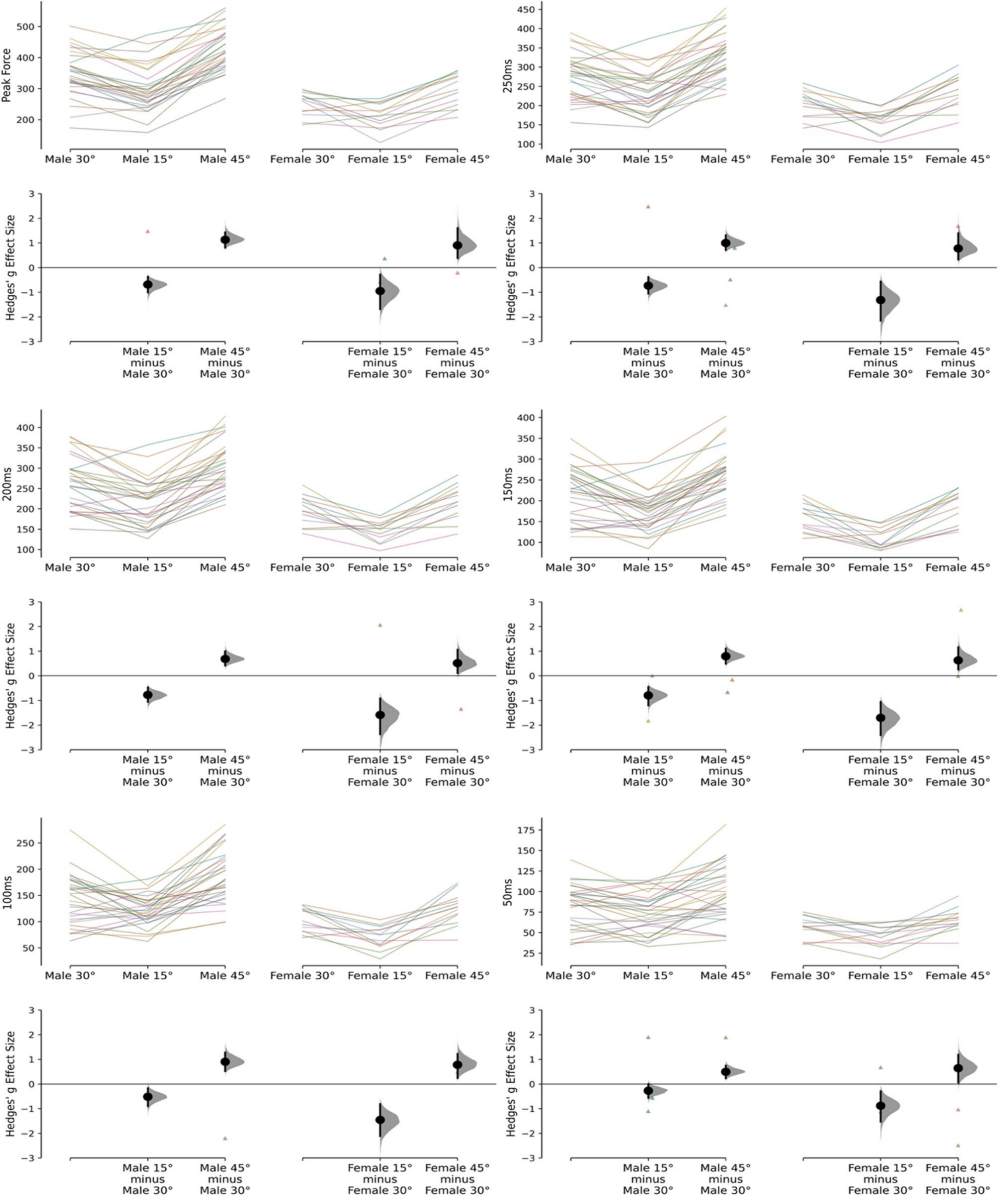
Cummings estimation plots illustrating paired comparisons and bootstrapped effect sizes for net force output across knee flexion angles (30°, 15°, and 45°) at multiple force–time points, presented separately for male and female participants

All statistical analyses were performed using Python 3.12.7 (Python Software Foundation, DE, USA). Data are reported as mean ± SD. The Shapiro-Wilk test was used to evaluate normality and the Levene’s test to assess homogeneity of variance. An a priori threshold of α < 0.05 was set for all analyses. Within-session absolute reliability was calculated using coefficient of variation (CV%) based on the sample SD and 95% confidence intervals (CI) as < 5.00% (excellent), 5.00–9.99% (good), 10.00–14.99% (moderate), and >15.00% (poor). Within-session relative reliability was calculated using two-way absolute agreement intraclass correlation coefficients (ICC; 3,1) and interpreted based on the lower bound CI as >0.90 (excellent), 0.75–0.89 (good), 0.50–0.74 (moderate), and < 0.49 (poor) [29]. To assess force at specific time points (50 ms, 100 ms, 150 ms, 200 ms, 250 ms, and peak force) across different knee angles (15°, 30°, and 45°), a series of one-way repeated measures analysis of variance (RM-ANOVAs) were conducted. If the Levene’s test for equality of variance was violated, a Welch’s ANOVA was used instead. Partial eta squared (*η*_*p*_*²*) was calculated to quantify the variance explained by the knee angle and interpreted as ≥ 0.01 (small), ≥ 0.06 (moderate) and ≥ 0.14 (large) as indicated by Yagin et al. [30]. Bonferroni post hoc analyses were conducted to account for multiple comparisons while Hedges’ g effect sizes were performed for all pairwise comparisons and interpreted according to Hopkins’ [31] thresholds: ≤0.19 (trivial), 0.20–0.59 (small), 0.60–1.19 (moderate), 1.20–1.99 (large), and 2.00–3.99 (very large). Cumming estimation plots are used to visualize the data, displaying individual and paired values as bootstrapped sample distributions with 95% CI (Fig. 2).

## Results

Good to excellent absolute and relative reliability was observed for net peak force, force at 200- and force at 250 ms across both males and females (see Table 1). Earlier time points (50–150 ms) demonstrated mixed relative reliability (moderate to good for males and poor to good for females) and absolute reliability (poor to moderate for both sexes), with a general trend of improving reliability at later time points. Significant differences in force production were observed between knee angles and across all force-time points (50 ms, 100 ms, 150 ms, 200 ms, 250 ms, and peak force) for both males and females (*p* < 0.05), with force progressively increasing over time and with greater knee angles. Partial eta squared values indicated a large effect of knee angle on force production, with variance accounted for by knee angle ranging between 0.30 and 0.82 (male *η*_*p*_*²*), and 0.52–0.76 (female *η*_*p*_*²*; see Table 2). Results of post hoc analyses revealed the 45° knee angle produced greater force than the 30° and 15° knee angle, and the 30° angle produced greater force than the 15° knee angle (*p* < 0.05) across all force-time points. Pairwise comparisons revealed effect sizes ranging between small-large for males (*g* = 0.27–1.84) and moderate to very large for females (*g* = 0.51–2.18) across individual force-time points.

**Table 1:** Within-session relative (ICC) and absolute (CV) reliability with 95% confidence intervals

Variable	15°		30°		45°	
	ICC (95% CI)	CV (95% CI)	ICC (95% CI)	CV (95% CI)	ICC (95% CI)	CV (95% CI)
Males						
Net Force at 50 ms	0.81 (0.66, 0.90)	25.67 (21.10, 30.24)	0.82 (0.66, 0.90)	23.74 (19.62, 27.85)	0.86 (0.74, 0.93)	26.10 (21.75, 30.46)
Net Force at 100 ms	0.73 (0.52, 0.86)	21.82 (17.39, 26.25)	0.86 (0.74, 0.93)	20.08 (16.22, 23.93)	0.84 (0.70, 0.92)	18.54 (14.56, 22.51)
Net Force at 150 ms	0.82 (0.67, 0.91)	15.58 (13.10, 18.06)	0.91 (0.84, 0.96)	12.64 (9.45, 15.83)	0.88 (0.78, 0.94)	10.81 (8.73, 12.88)
Net Force at 200 ms	0.89 (0.80, 0.94)	10.88 (8.85, 12.91)	0.95 (0.91, 0.97)	8.50 (7.09, 9.92)	0.89 (0.81, 0.95)	8.05 (6.85, 9.25)
Net Force at 250 ms	0.92 (0.85, 0.96)	9.07 (7.61, 10.53)	0.97 (0.94, 0.98)	7.14 (5.86, 8.42)	0.91 (0.84, 0.96)	8.06 (7.06, 9.06)
Net Peak Force	0.99 (0.97, 0.99)	6.10 (5.06, 7.14)	0.99 (0.97, 0.99)	4.85 (4.07, 5.63)	0.98 (0.96, 0.99)	3.69 (2.98, 4.40)
Females						
Net Force at 50 ms	0.73 (0.32, 0.91)	28.76 (22.47, 35.04)	0.72 (0.31, 0.90)	26.40 (19.90, 32.90)	0.42 (−0.47, 0.81)	26.72 (20.60, 32.85)
Net Force at 100 ms	0.92 (0.79, 0.97)	20.81 (13.76, 27.85)	0.83 (0.57, 0.94)	17.48 (11.95, 23.02)	0.72 (0.29, 0.91)	26.67 (17.40, 35.95)
Net Force at 150 ms	0.88 (0.70, 0.96)	20.98 (10.82, 31.14)	0.92 (0.80, 0.97)	11.16 (8.24, 14.07)	0.86 (0.65, 0.95)	14.17 (9.64, 18.71)
Net Force at 200 ms	0.92 (0.80, 0.97)	10.00 (7.89, 12.11)	0.97 (0.93, 0.99)	9.60 (6.68, 12.51)	0.96 (0.91, 0.99)	8.77 (6.62, 10.92)
Net Force at 250 ms	0.92 (0.80, 0.97)	8.83 (6.66, 10.99)	0.97 (0.92, 0.99)	8.14 (4.86, 11.41)	0.95 (0.88, 0.98)	8.64 (6.14, 11.14)
Net Peak Force	0.98 (0.95, 0.99)	6.16 (4.55, 7.77)	0.99 (0.96, 0.99)	4.54 (3.55, 5.54)	0.99 (0.98, 1.00)	3.92 (2.76, 5.08)

*ICC* intraclass correlation coefficient, *CV%* coefficient of variation percentage, *CI* confidence interval

**Table 2:** Comparison of outputs across the three knee angles

Variable	15° (Mean ± SD)	30° (Mean ± SD)	45° (Mean ± SD)	Partial ETA Squared (ηₚ²)	Hedges’ g
Males					
Net Force at 50 ms (N)	75.4 (23.2)	81.8 (26.8)	98.3 (35.3)	0.82	0.69;1.84
Net Force at 100 ms (N)	120.4 (27.7)	142.8 (48.4)	188.5 (52.5)	0.72	0.73;1.79
Net Force at 150 ms (N)	175.4 (45.9)	222.1 (64.8)	269.8 (57.0)	0.68	0.74;1.61
Net Force at 200 ms (N)	217.0 (56.5)	264.8 (66.7)	307.7 (58.8)	0.69	0.80;1.82
Net Force at 250 ms (N)	235.3 (54.7)	278.1 (60.5)	337.1 (60.4)	0.30	0.52;1.59
Net Peak Force (N)	299.6 (71.8)	350.8 (75.5)	428.0 (69.1)	0.36	0.27;0.53
Females					
Net Force at 50 ms (N)	46.6 (10.4)	57.2 (12.7)	65.4 (12.9)	0.65	0.90;1.67
Net Force at 100 ms (N)	71.2 (19.3)	101.8 (22.9)	124.1 (28.0)	0.70	0.77;1.99
Net Force at 150 ms (N)	108.6 (25.4)	156.1 (36.4)	183.6 (37.6)	0.72	0.51;1.98
Net Force at 200 ms (N)	145.6 (26.1)	190.9 (40.9)	216.4 (39.3)	0.76	0.63;2.18
Net Force at 250 ms (N)	161.3 (28.8)	199.8 (41.2)	237.0 (41.3)	0.71	0.78;2.06
Net Peak Force (N)	212.7 (40.9)	243.9 (46.7)	292.5 (50.2)	0.52	0.64;1.53

*SD* standard deviation, *ηₚ²* Partial eta squared

## Discussion

The aim of this study was to determine the impact of knee angle variability on force production outcomes during the isometric single-leg long lever bridge to determine testing rigor requirements and ensure standardized testing procedures are consistently followed for accurate and reproducible results. The main findings reveal substantial contributions to force production due to knee angle variability during the assessment set-up. In previous isometric posterior chain assessments performed with portable fixed dynamometry, large variations have been seen either due to various testing procedures or changes in knee angle during similar protocols [2]. Similarly, the isometric long-lever bridge displays excessively large variations in force production as determined by the large effect sizes in knee angle variations. Additionally, the pairwise comparisons of knee angles between 15–30° and 30–45° in males, showing small to large effect sizes, along with the comparisons in females demonstrating moderate to very large effect sizes, suggests a substantial impact on changes in knee angles as small as 15 degrees. Surprisingly, for males, larger pairwise differences with moderate effect sizes were observed between the 30–45° knee angle, compared with small to moderate effect sizes observed between the 15–30° knee angle. In contrast, females exhibited the opposite pattern, displaying moderate to large effect sizes between the 15–30° knee angle and moderate effect sizes between the 30–45° knee angle. This suggests that the male and female population may differ in strategies when maximizing force output during the long-lever bridge assessment at various knee angles. Given that rapid force production appears relatively consistent between sexes when normalized to peak force, the observed sex-based differences in angle sensitivity may be linked more closely to disparities in relative strength. However, while females in this sample exhibited slightly larger absolute percent increases in force output across angles, particularly from 15° to 30°, normalizing to body mass did not substantially alter this pattern. In both males and females, the percentage increase between knee angles remained nearly identical after normalization, suggesting that relative strength alone does not fully explain these differences. This implies that other factors may play a more prominent role in modulating force output across joint angles. Broadly, anatomical and physiological considerations such as limb segment lengths or fiber-type composition, which might differ between males and females due to sport-specific backgrounds, could influence force production across joint angles and warrants further investigation. Further research should explore how these elements interact, particularly in the context of long-lever bridge assessments. Moreover, large differences were observed between the 15–45° knee angles for the male population and large to very large differences for the female population. As expected, larger variations in knee angle led to greater observed differences in force production. Resultingly, rigid knee angles should be meticulously executed when performing the long-lever bridge as minor variations may lead to moderate to large changes in force production and greater variations in knee angle may result in large to very large changes. As such, unwanted or excessive movement between repetitions or between limbs must be thoroughly assessed during each trial to ensure consistent and reliable results and avoid any unwanted noise due to lack of testing rigor. Practitioners can achieve this by visually assessing each repetition and enforcing a strict protocol, preferably employing measurement tools, such as a standard goniometer to measure joint movement more precisely, and repeating trials when deviations occur, thereby minimizing noise and ensuring reliable results.

In relation to various isometric testing procedures, mixed results have been displayed with either no effect due to joint angle changes [32], or large differences depending on force-time points [33]. Large variations in torque have been observed due to changes in knee joint angles during isometric knee extension tasks [34], and knee flexor tasks [35], specifically when looking at rapid force generation. In concordance, the results of the current study display large variations in force production, with force increasing at greater knee angles, indicating the importance of controlling knee angles to mitigate variance in force output. Similarly, relationships have been observed across a variety of multi-joint assessments, with certain tests increasing force outputs at longer lever lengths due to increased mechanical advantage such as the isometric back squat [36], isometric belt squat [37], and isometric leg press [38]. Measurements such as muscle activation [39] and force output [25] may both be highly dependent on the various joint angles performed, across a variety of movement tasks, however, despite the overall increase in ground reaction forces, the muscular force contributions from the BF_LH_ may be reduced [40], thus minimizing the relevance for evaluating the intended musculature of the assessment. As such, despite the application of the long-lever bridge being most applicably utilized as a field-based assessment, the rigorous calculation of knee joint angles must be thoroughly controlled to ensure the test accurately targets the intended musculature and provides relevant information regarding intended muscular force outputs. Future research should investigate how variations in joint angle influence muscle-specific activation during this assessment, particularly in relation to the BF_LH_ to improve its specificity and diagnostic utility.

When considering long-term measurements examining various adaptations and potential chronic fatigue due to high training loads, the importance of ensuring a rigorous testing procedure becomes even further important. Pairwise comparisons from periodic evaluations, such as pre- and post-tests must be consistent to ensure reliable measurements and accurate results. Furthermore, individual variations and test-retest comparisons may be compromised due to poor testing rigor, leading to inconsistencies between assessments. This potential lack of consistency would make it increasingly difficult to measure true changes caused by longitudinal neuromuscular adaptations. If the procedures are not rigorously controlled, any observed changes may be falsely attributed to training effects or fatigue when they result in reality from methodological inconsistencies. Such error could lead to faulty assumptions regarding the success or failure of an intervention, whether that be a training intervention or recovery-based intervention, ultimately misguiding decisions and limiting the ability to draw accurate conclusions. Furthermore, while the immediate effects may not be severe, repeated misguidance could influence intervention strategies, potentially resulting in excessive recovery periods that may hinder training progress and, over time, contribute to detraining. Moreover, between group comparisons may be further troublesome due to the lack of standardization as performance-based assessments are often used to provide benchmark values and or assess individual athletes in comparison to team norms. Such interest could also be difficult to execute with lack of standardization as reference values may not accurately reflect the true team performance and normative values may not be correctly calculated. In turn, valid assessment of players progress or potential need for improvement through assessing performance-limiting factors could be challenging which may play a vital role in sustaining long-term athletic performance and success. 

Identifying an athlete’s weaknesses or strengths, guiding intervention strategies and evaluating performance in rehabilitation-based programs could be considered the cornerstone of sports performance monitoring [41], but may prove challenging with variance controlled for by as high as 80%. As such, the rigor of the testing set up must be controlled as thoroughly as possible while ensuring minimal variability due to altered knee joint angles. In agreement with Read et al. [21], for general standardization, a knee flexion angle of 30° is recommended. This aligns with previous research where several studies have employed 30° to ensure comparability and reproducibility in isometric posterior chain assessments [4, 5, 13, 26, 42]. Other angles (e.g., 15° or 45°), may be useful for specific testing purposes, such as evaluating extreme ranges of motion or emphasizing the medial hamstring musculature, respectively [22, 24]. However, the end-range positions is associated with reduced reliability for early force-time points, particularly below 200 ms, meaning rapid force-time characteristics may not be accurately captured. Therefore, across both males and females, measurements at 50- and 100 ms should likely be avoided due to high measurement error, which could mask true performance and falsely influence longitudinal comparisons. On the other hand, later time points such as 200- and 250 ms as well as peak force demonstrate good to excellent reliability across both males and females. For the 30° and 45° knee angles, reliable measurements can generally be obtained from 150 ms onward, making these positions suitable for capturing rapid force characteristics without requiring extreme end ranges. Taken together, these findings support the selection of a 30° knee angle as a practical standard, as it combines high measurement reliability with moderate joint positioning, reduces overreliance on the medial hamstring musculature, and avoids extreme end ranges. As such, adopting 30° as a standard facilitates consistency in reporting and allows meaningful comparison across studies, while providing an appropriate middle ground.

### Limitations

It should be acknowledged that the present study is not without limitations. First, the measurements performed did not include techniques capable of assessing muscle activation or recruitment patters. The primary aim was therefore to examine differences in overall force production across knee joint angles. While this approach was informed by prior research suggesting differential muscle activation across various knee joint angles, this study does not provide evidence towards isolating individual hamstring musculature. Rather, it examines force production at angles where prior research indicates preferential recruitment, with the theoretical premise that such an approach may allow greater insight and more informed decision making regarding HSI risk factors than that of other assessed knee joint angles. Moreover, the potential and likely involvement of other musculature, such as the hip extensors in addition to synergistic muscles involved in knee flexion, cannot be excluded. As such, while the testing protocol was designed with the hope of preferential recruitment at specific joint angles, no empirical evidence, either from the present study or from prior research has been collected to confirm this. Therefore, the measured force should be interpreted as the combined output of multiple muscle groups rather than being attributed exclusively to the BF_LH_ or the hamstring musculature as a whole. Finally, it is worth noting that the standardization of hip positioning during the assessments may have influenced force production in ways not directly evaluated in the present study. Variations in individual torso length, for instance, could result in subtle differences in hip joint angles despite the standardized setup, which may partially explain the variability observed in the measured force outcomes.

## Conclusion

The single-leg long-lever bridge is sensitive to knee angle variations, emphasizing the need for strict control of joint positioning to ensure consistent monitoring outcomes in high-performance environments. The variations in force production across different knee angles and time points further emphasize that meaningful longitudinal monitoring cannot be achieved without rigorous standardization of joint positioning through testing. To emphasize the musculature of the BF_LH_, lower knee angles should be applied to prevent a reduced focus of the target musculature. Consequently, to further improve consistency between assessments, rigorous testing procedures should be applied and continuously reassessed between repetitions to ensure standardized protocols and accurate measurements are being performed to allow for proper evaluations. Standardizing the long-lever bridge testing protocol to accurately assess the intended musculature will reduce unintended variability and improve consistency between measures, thereby informing practitioners in high-performance, rehabilitation, medical, and related fields of meaningful monitoring outcomes.

## Data Availability

The data that support the findings of this study are available from the corresponding author upon reasonable request.
